# Caffeic acid inhibits *Staphylococcus aureus*‐induced endometritis through regulating AMPKα/mTOR/HIF‐1α signalling pathway

**DOI:** 10.1111/jcmm.70175

**Published:** 2024-10-27

**Authors:** Lu Cao, Junbao Liu, Cong Ye, Yubo Hu, Rui Qin

**Affiliations:** ^1^ Department of Obstetrics China‐Japan Union Hospital of Jilin University Changchun Jilin China; ^2^ Department of Gynecology China‐Japan Union Hospital of Jilin University Changchun Jilin China; ^3^ Department of Anesthesiology China‐Japan Union Hospital of Jilin University Changchun Jilin China

**Keywords:** AMPK, endometritis, ferroptosis, inflammation, *Staphylococcus aureus*

## Abstract

Endometritis is mostly caused by childbirth or postpartum uterine infection. It is one of the important reasons leading to female infertility. Caffeic acid (CA) and its derivatives are widely found in some foods and traditional Chinese medicine, and have biological activities such as antioxidant, free radical scavenging, anti‐inflammatory, and anti‐infection. In this study, we aimed to explore the effect of CA on *Staphylococcus aureus*‐induced endometritis. The contents of TNF‐α and IL‐1β were detected by ELISA in *S. aureus*‐induced endometritis model. Western blot assay was used to detect the expression of AMPKα/mTOR/HIF‐1α pathway related proteins and GPX4 expression. In addition, the concentrations of MDA, GSH, and iron were tested by the assay kits. Compared with the model group, CA treatment significantly alleviated *S. aureus*‐induced uterine injury, MPO activity, the contents of inflammatory factors TNF‐α and IL‐1β, and NF‐κB activation. Meanwhile, CA significantly inhibited *S. aureus*‐induced ferroptosis, as confirmed by decreased MDA and iron concentration and up‐regulated GPX4 expression and GSH level. Furthermore, CA attenuated *S. aureus*‐induced HIF‐1α and phosphorylated mTOR expression and increased phosphorylated AMPK expression. In conclusion, CA inhibits inflammation and ferroptosis by regulating AMPKα/mTOR/HIF‐1α signalling pathway to alleviate *S. aureus*‐induced endometritis in mice.

## 1 INTRODUCTION

Endometritis is inflammation caused by bacteria breaking through the defence of the cervix and invading the endometrium.^1^ If not treated in time, the inflammation can easily spread and cause uterine serositis, which can become chronic inflammation, disrupt the estrus cycle, cause reproductive disorders, and ultimately lead to long‐term infertility in human and livestock, causing huge economic losses to the development of the breeding industry.^2^ There are various reasons for the occurrence of endometritis, mainly related to the infection of pathogenic microorganisms. *Staphylococcus aureus* is the main bacteria causing endometritis and it enters the uterus through various channels and multiply in large numbers, causing inflammation of the uterine mucosa.^3^ During the development of endometritis, *S. aureus* can induce neutrophil activation, leading to the release of inflammatory cytokines and ultimately endometrial damage.^4^ Numerous studies have shown that inhibiting inflammatory cytokines has a potential therapeutic effect on endometritis.^5^ At present, commonly used methods for treating endometritis include irrigation, antibiotics, hormones, and so forth, but there are problems such as unsatisfactory treatment effects, drug resistance, and drug residue.^6^, ^7^ Therefore, screening natural and low side effect candidate drugs for the prevention and treatment of endometritis in human and livestock can provide a solution for clinical prevention and treatment of endometritis.

Ferroptosis is a regulatory cell death mechanism based on iron driven lipid peroxidation, involved in the pathological processes of various diseases.^8^ Ferroptosis can serve as a therapeutic target for many diseases.^9^ Recent studies demonstrated that ferroptosis was involved in the development of endometritis.^10^ And inhibition of ferroptosis could exhibit protective roles against endometritis. AMPK is a conserved cellular energy receptor that plays an important role in regulating cell growth, proliferation, differentiation, and other aspects.^11^ The AMPK related signalling pathway is an important pathway for regulating ferroptosis.^12^ Targeting AMPK to regulate ferroptosis will provide new ideas for future disease treatment and new targets for new drug research.

Caffeic acid (CA) and its derivatives are commonly found in some foods and traditional Chinese medicine, and are widely distributed secondary metabolites in plants.^13^ Research has shown that CA and its derivatives are natural fungicides that can inhibit various fungal and bacterial infections.^14^ Mishra RK et al. indicated that inflammation is reduced by a nanomicelle conjugated with CA in experimental colitis.^15^ Yu CM et al. proved that CA could alleviate sepsis‐induced organ injury through inhibiting inflammation and neutrophil activation.^16^ Moreover, LPS‐mediated inflammation in mammary tissue is alleviated by CA treatment.^17^ In addition, CA exhibits a protective role against intestinal injury through attenuating inflammation and regulating gut microbiota.^18^ However, no studies have been reported on the effects of CA on endometritis. The aim of this study was to investigate the effects of CA on endometritis and clarify its mechanisms.

## 2 MATERIALS AND METHODS

### 2.1 Materials

CA (purity>98%) was supplied by Sigma (CA, USA). ELISA kits were obtained from Biolegend (San Diego, CA, USA). The AMPKα, mTOR, HIF‐1α, GPX4, and NF‐κB signalling pathway antibodies used in this study were obtained from CST (MA, USA).

### 2.2 Animals and experimental design

Female C57 mice weighing about 20–25 g on average were obtained from Liaoning Changsheng Biotechnology Co., Ltd. (Benxi, China). The mice were divided into five groups and each group contained 12 mice: control group, *S. aureus* group, CA (10, 20, 30 mg/kg) + *S. aureus* groups. The *S. aureus*‐induced endometritis mouse model was developed as follows.^19^ Mice were administered equal amounts of *S. aureus* on each side of the uterus. CA (10, 20, 30 mg/kg) was given to mice in each group by intraperitoneal injection. 24 h later, the uterine tissues from each group were harvested for subsequent studies. All studies were approved and consented by the Animal Use Committee of Jilin University.

### 2.3 Histological analysis

Uterine tissue samples for histological analysis were fixed in 4% paraformaldehyde for 48 h and then embedded in paraffin to prepare 5 μm paraffin sections. All sections were dewaxed hydrated and stained with haematoxylin and eosin (H&E). Finally, the uterine histological changes were observed using optical microscopy.

### 2.4 Cytokine assay by ELISA

Uterine tissue was collected and prepared as 10% homogenates using PBS. After centrifuging at 12000 × g for 10 min, the supernatants were obtained. Then, TNF‐α and IL‐1β production was tested by the ELISA kits (Biolegend, USA). The optical density of each well was read at 450 nm.

### 2.5 Western blotting

Total protein from uterine tissue was harvested using tissue protein extracts (Thermo, USA). Proteins (30 μg) were separated using 12% SDS‐PAGE and then incorporated with 0.45 μm PVDF membranes. After sealing in 5% skim milk, the prepared PVDF membranes were incubated overnight at 4°C with specific primary antibodies, including GPX4, AMPK, mTOR, and NF‐κB. After washing three times with TBST, PVDF membranes were treated with goat anti‐rabbit or rabbit anti‐mouse IgG (1:20,000, Affinity Biosciences, OH, USA) for 2 h at room temperature. Finally, proteins were determined by ECL plus protein blotting detection system (Tanon, China).

### 2.6 Ferroptosis detection

The concentrations of MDA, GSH, and iron were measured by the assay kits according to the instructions. GPX4 and xCT expression was detected by western blot analysis.

### 2.7 Statistical analysis

GraphPad Prism 9.5 was used for the statistical analysis. Data are expressed as boxplots or the mean ± SD. Significant differences were evaluated using one‐way analysis of variance (ANOVA) followed by Tukey's test. *p* < 0.05 indicates significant difference.

## 3 RESULTS

### 3.1 Effects of CA on *S. aureus*‐induced uterine pathological damage

**FIGURE 1:**
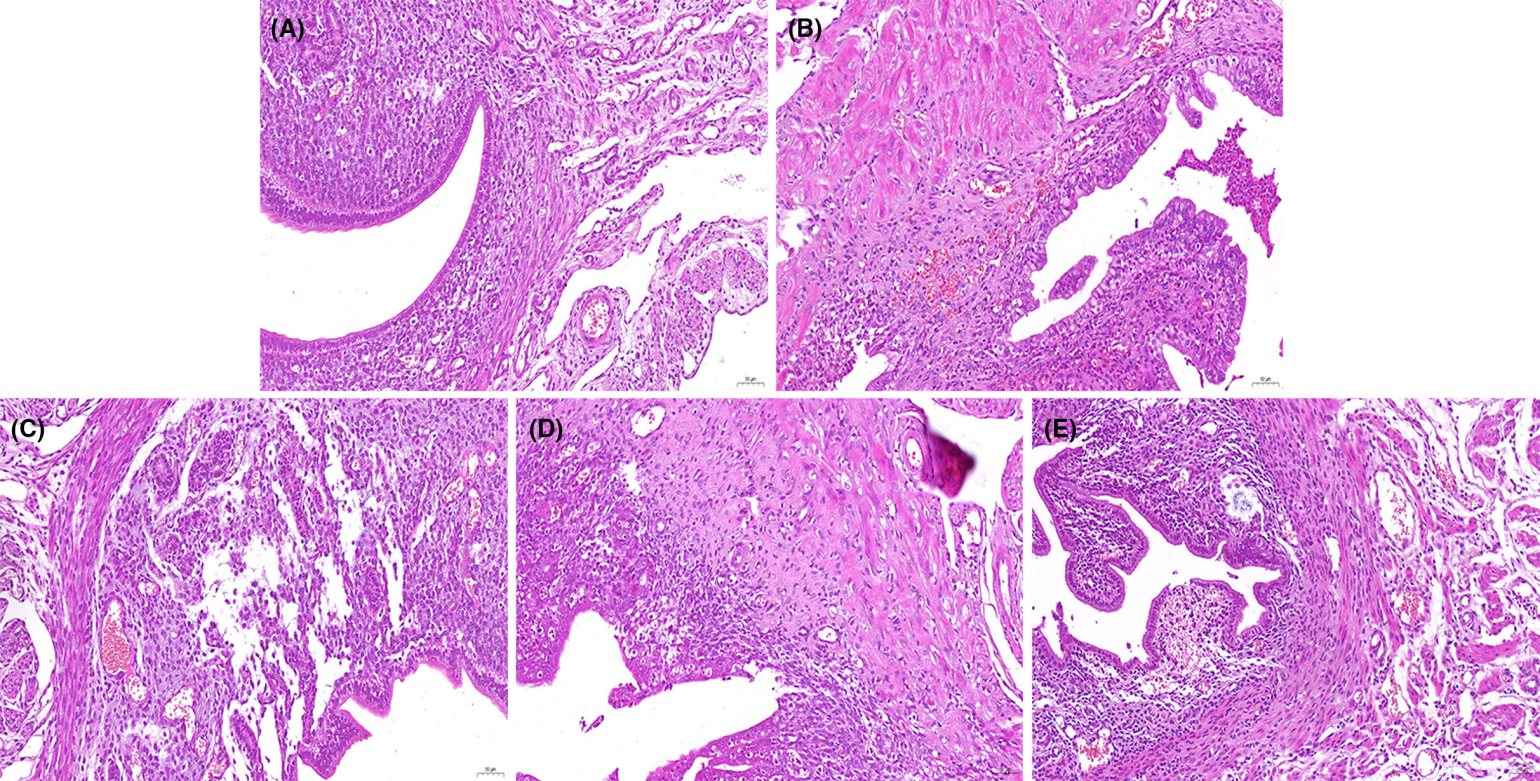
Effects of CA on *Staphylococcus aureus*‐induced uterine histopathological changes. Histopathologic sections of uterine tissues (H&E, × 100). (A) control, (B) CA (30 mg/kg) group, (C) *S.aureus* group, (D–E) CA (10, 20, 30 mg/kg) + *S.aureus* groups.

H&E staining is applied to assess the protective capacity of CA on *S. aureus*‐induced uterine injury. Uterine tissues of *S. aureus* group demonstrated severe histopathologic changes, including epithelial cell abscission, injury, and inflammatory cell infiltration. However, *S. aureus*‐induced histopathologic injuries were markedly prevented by CA (Figure 1).

#### 3.1.1 CA attenuates *S. aureus*‐induced TNF‐α and IL‐1β production

**FIGURE 2:**
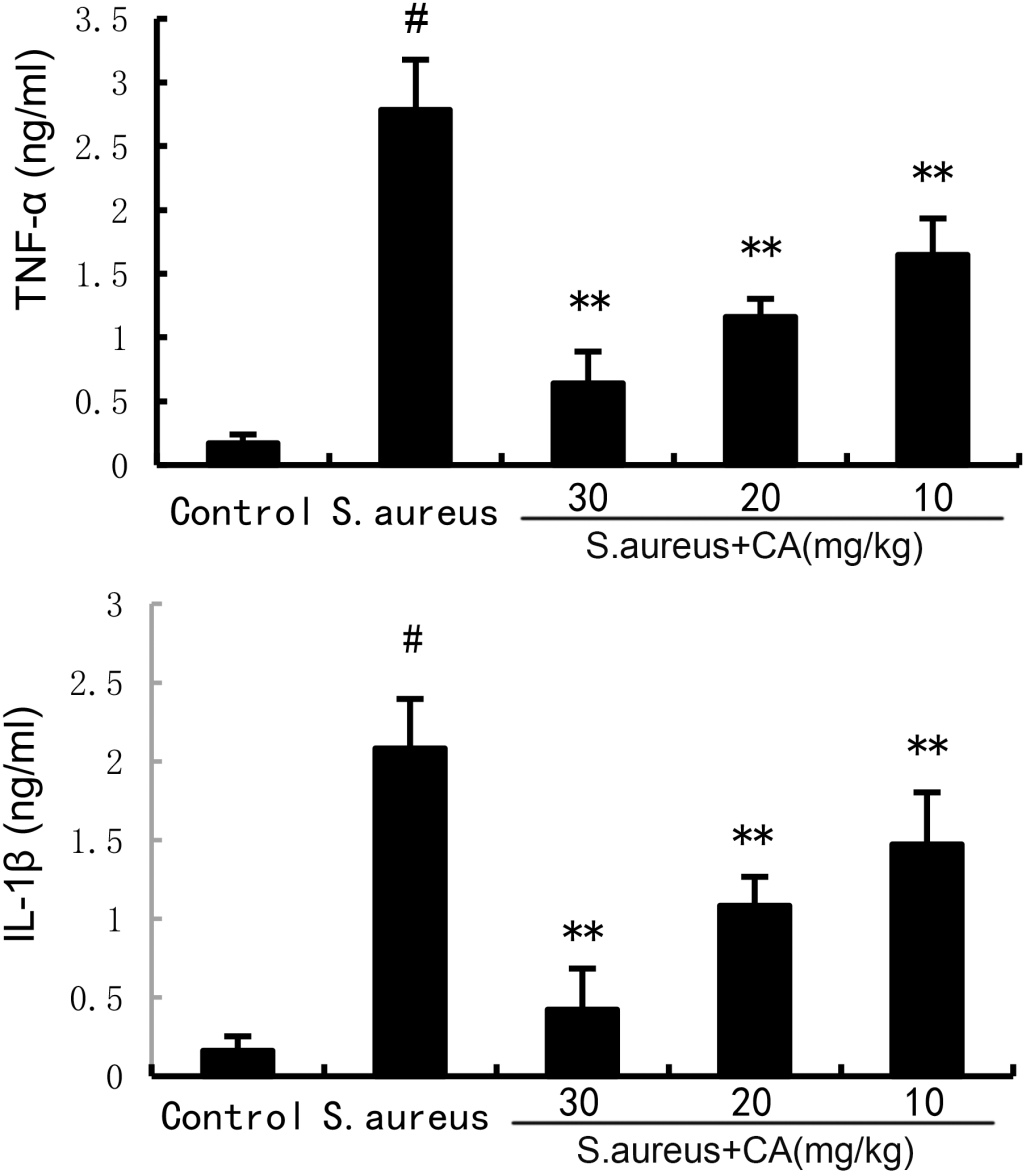
Effect of CA on inflammatory cytokine production. The values presented are the mean ± SD. ^#^ *p* < 0.01 is significantly different from control group; **p < 0.01 are significantly different from *Staphylococcus aureus* group.

ELISA results showed a marked increase in TNF‐α and IL‐1β production at 24 h post *S. aureus* administration. However, after administration of CA, TNF‐α and IL‐1β production was markedly decreased (Figure 2).

### 3.2 CA alleviates MPO activity induced by *S. aureus*

**FIGURE 3:**
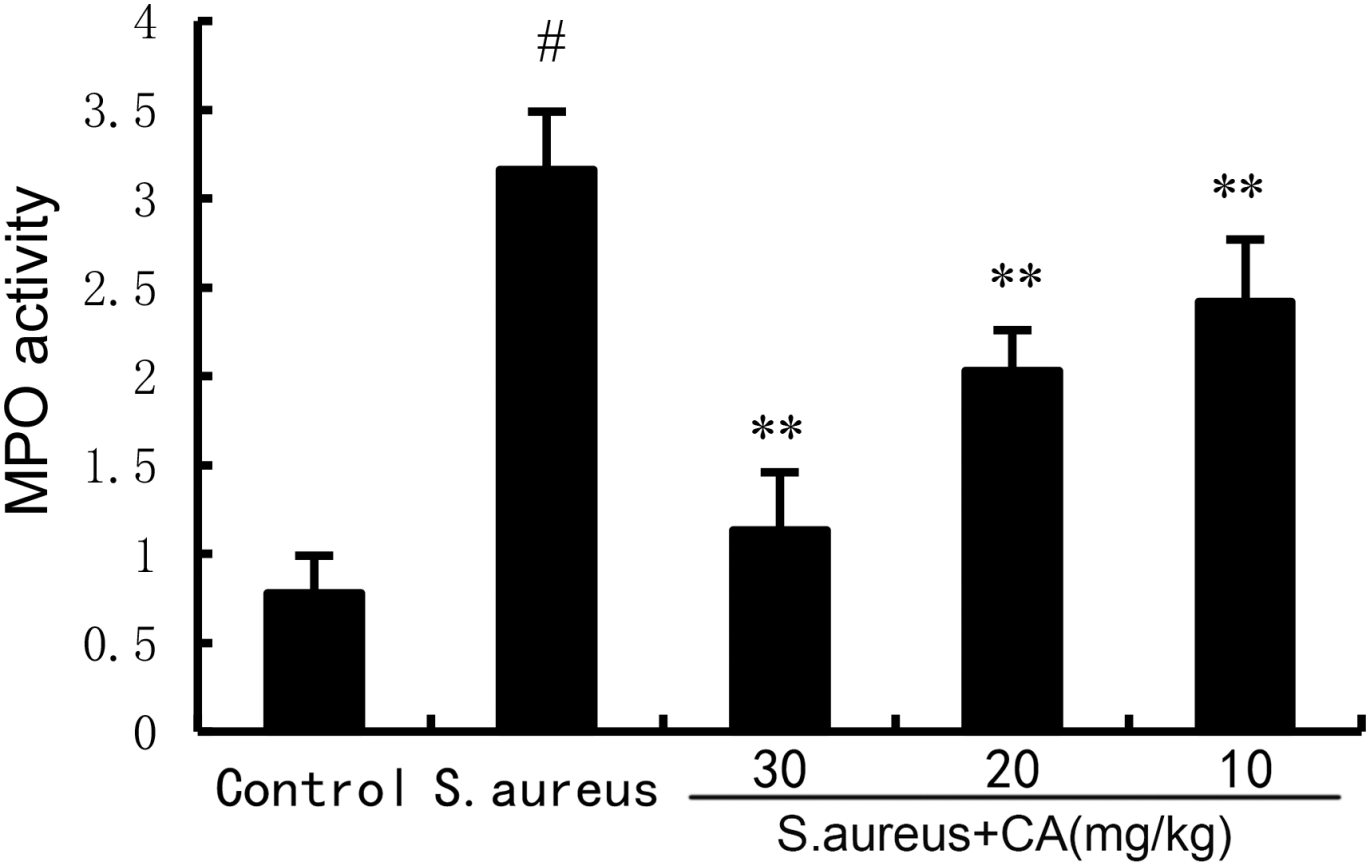
Effect of CA on MPO activity. The values presented are the mean ± SD. ^#^ *p* < 0.01 is significantly different from control group; **p < 0.01 are significantly different from *Staphylococcus aureus* group.

MPO activity was detected to assess the infiltration of neutrophils in uterine tissues. The results showed a marked increase in MPO activity at 24 h post *S. aureus* administration. However, after administration of CA, MPO activity was markedly attenuated (Figure 3).

#### 3.2.1 CA inhibits *S. aureus*‐induced ferroptosis

**FIGURE 4:**
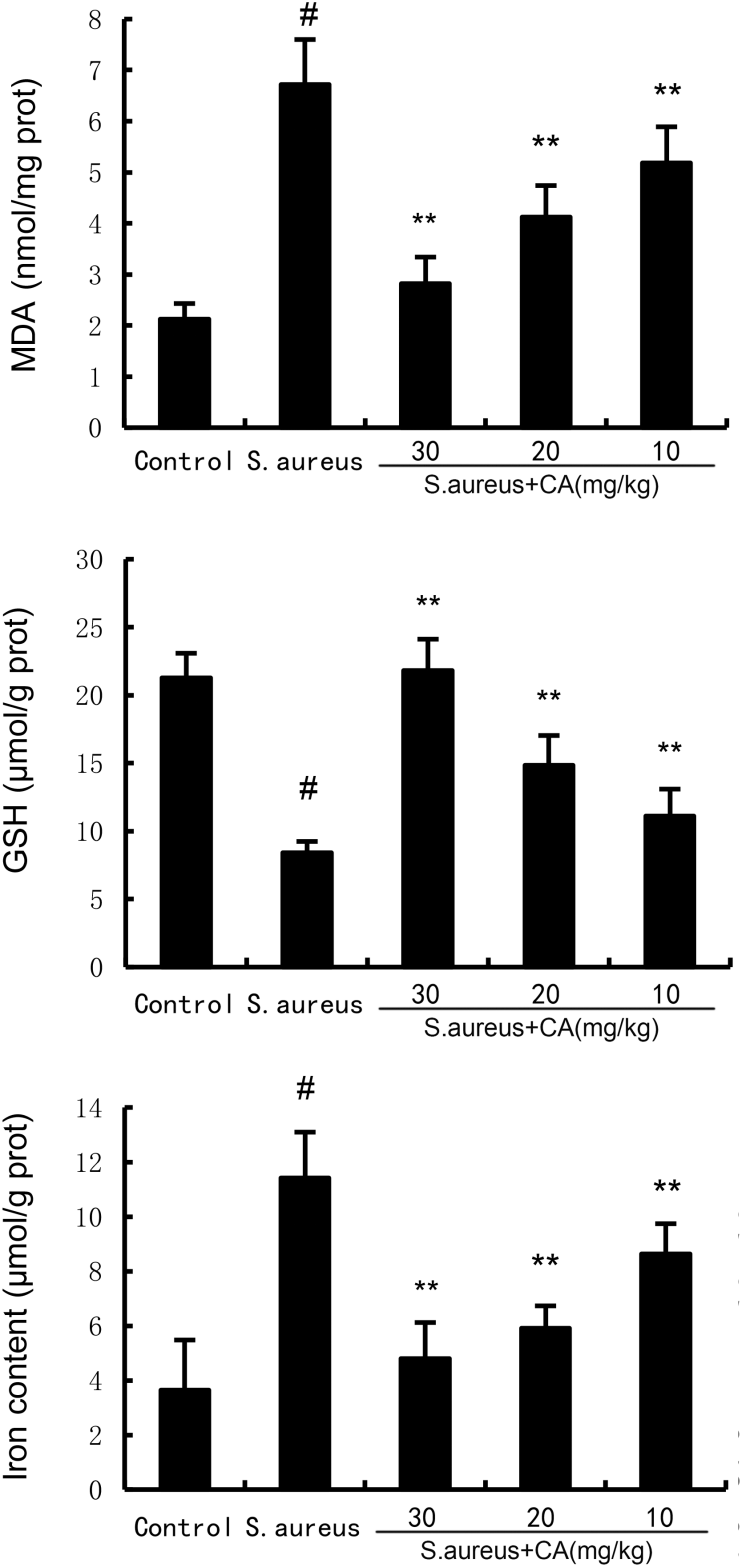
Effect of CA on GPX4 and ferritin expression. The values presented are the mean ± SD. ^#^ *p* < 0.01 is significantly different from control group; **p < 0.01 are significantly different from *Staphylococcus aureus* group.

**FIGURE 5:**
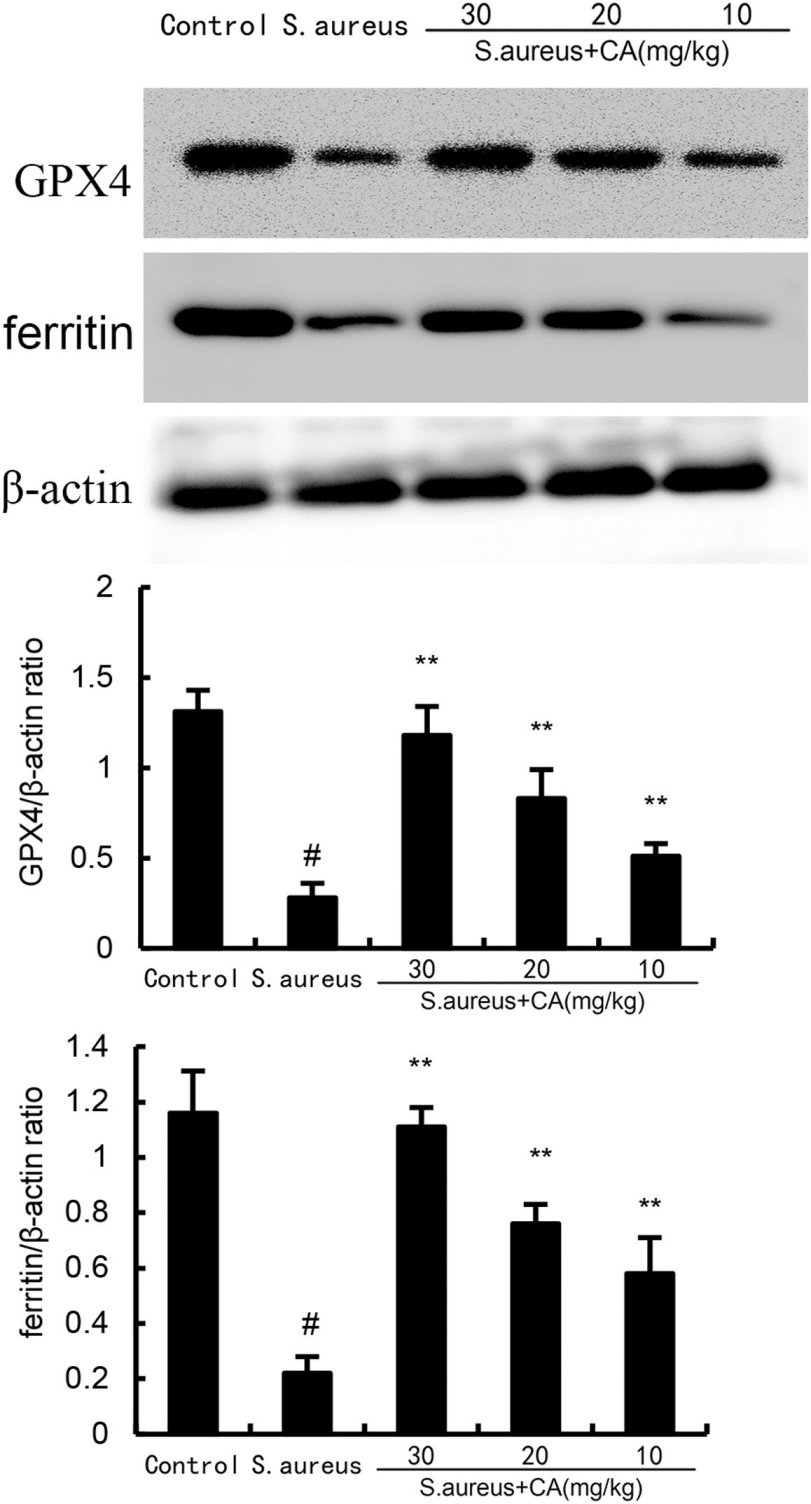
Effect of CA on MDA, iron, and GSH production. The values presented are the mean ± SD. ^#^ *p* < 0.01 is significantly different from control group; **p < 0.01 are significantly different from *Staphylococcus aureus* group.

Ferroptosis was assessed by measuring MDA, GSH, and Fe^2+^ production. The results showed a significant increase in MDA and Fe^2+^ production at 24 h post *S. aureus* administration. However, after administration of CA, MDA and Fe^2+^ production was markedly attenuated (Figure 4). Meanwhile, the data exhibited a significant decrease in GSH production and GPX4, xCT expression at 24 h post *S. aureus* administration. However, after administration of CA, GSH production and GPX4, xCT expression were markedly increased (Figure 5).

#### 3.2.2 CA attenuates *S. aureus*‐induced NF‐κB activation

**FIGURE 6:**
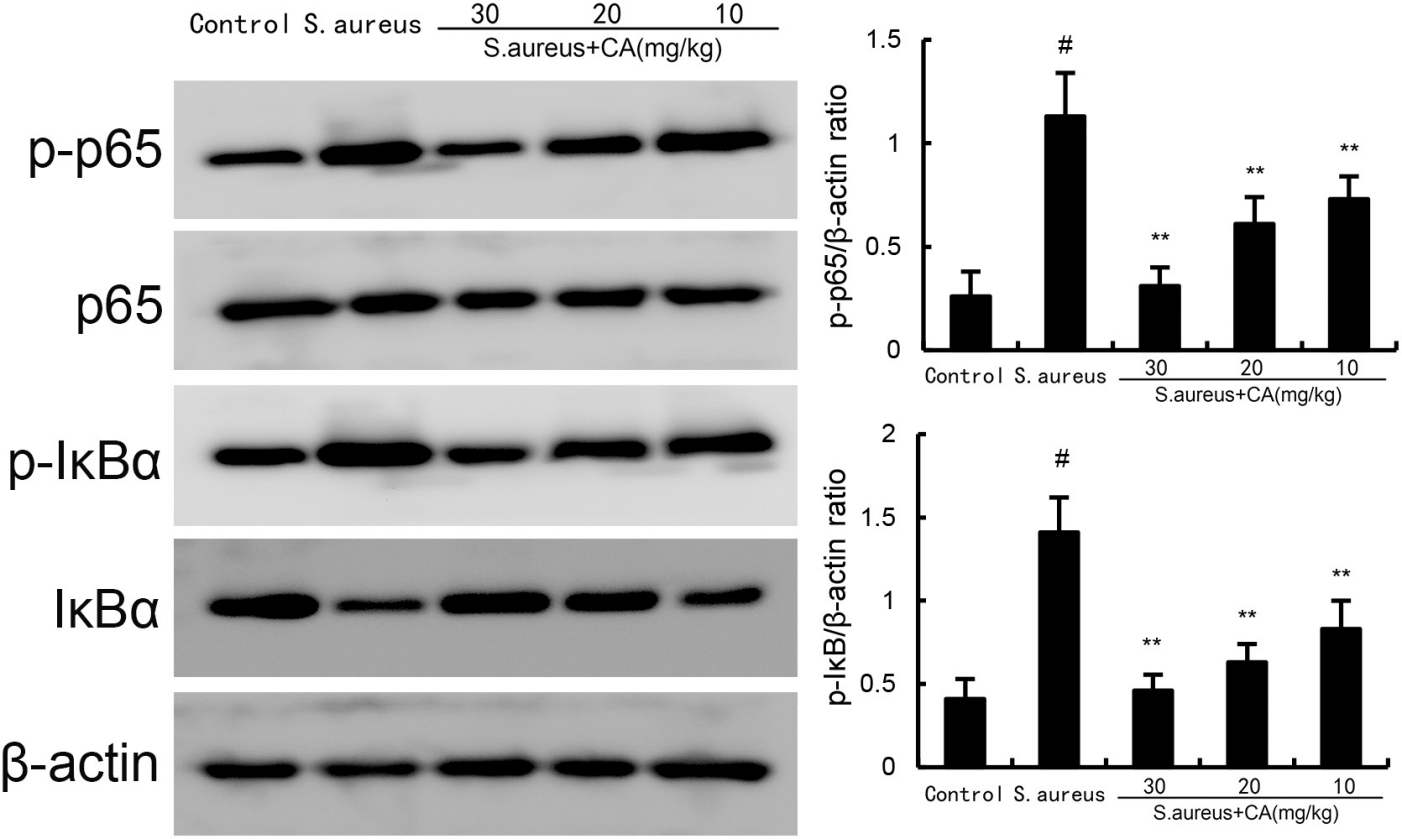
Effect of CA on NF‐κB activation in uterine gland. The values presented are the mean ± SD. ^#^ *p* < 0.01 is significantly different from control group; **p < 0.01 are significantly different from *Staphylococcus aureus* group.

NF‐κB signalling was tested to assess the anti‐inflammatory mechanism of CA. The data showed a marked increase in phosphorylated NF‐κB p65 and IκBα at 24 h post *S. aureus* administration. However, after administration of CA, phosphorylated NF‐κB p65 and IκBα expression was markedly attenuated (Figure 6).

### 3.3 Effects of CA on AMPKα/mTOR/HIF‐1α signalling pathway

**FIGURE 7:**
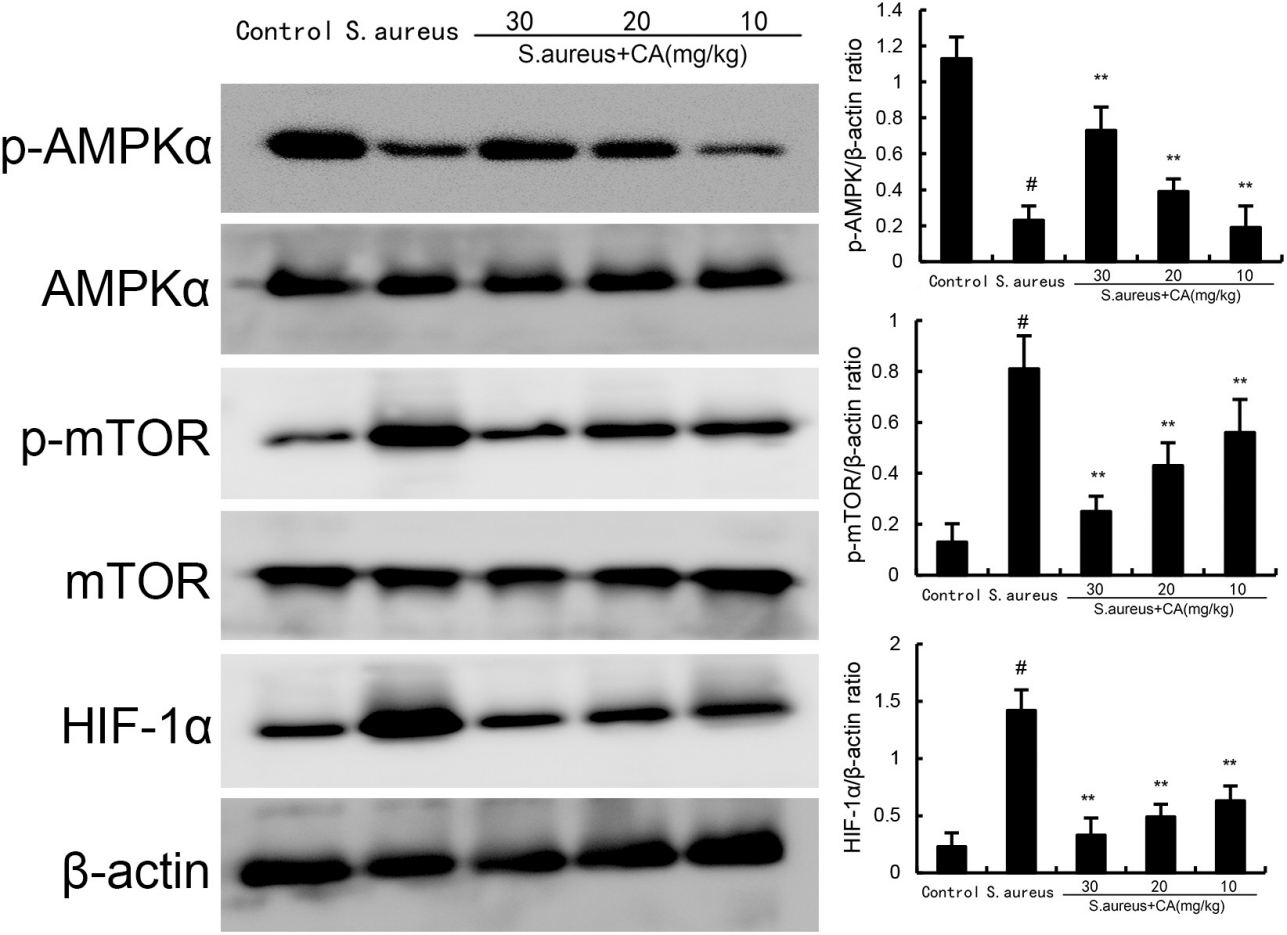
Effect of CA on AMPKα, mTOR, and HIF‐1α expression. The values presented are the mean ± SD. ^#^ *p* < 0.01 is significantly different from control group; **p < 0.01 are significantly different from *Staphylococcus aureus* group.

As Figure 6 displays, the mice in the *S. aureus* group exhibited increased HIF‐1α and phosphorylated mTOR expression and decreased phosphorylated AMPK expression. However, CA concentration‐dependently suppressed HIF‐1α and phosphorylated mTOR expression and increased phosphorylated mTOR expression (Figure 7).

## 4 DISCUSSION

Accumulating evidences demonstrated that specific inflammatory processes involved with the development of endometritis.^20^ However, the exact mechanisms of the progression of the disease are still being elucidated. Up till now, whether CA could inhibit the inflammation in the uterine has not been clarified. Our results showed that CA inhibited *S. aureus*‐induced endometritis through attenuating inflammatory response and ferroptosis.

Resistance to bacterial infection requires the body to drive the innate immune system to initiate a rapid and powerful inflammatory response, and the uterus as the first line of defence against bacterial infection, preventing the infection of pathogenic microorganisms, the endometrium plays an important role.^21^, ^22^ Studies demonstrated that increased uterine inflammation was observed in *S. aureus*‐induced endometritis.^23^ Increased TNF‐α and IL‐1β levels were found in uterine tissues of *S. aureus* treated mice.^19^ Nuclear transcription factor NF‐κB was initially discovered in B lymphocytes, and research has reported its role in regulating inflammation and immunity.^24^ In the classical signalling pathway of NF‐κB, the degradation of IκB protein leads to the release of NF‐κB dimer, which subsequently regulates the release of inflammatory cytokines.^25^ NF‐κB can be used as a target for endometritis treatment and inhibition of NF‐κB activation could alleviate uterine injury.^26^ In this study, CA inhibited *S. aureus*‐induced NF‐κB activation and inflammatory cytokine production.

Ferroptosis is a form of iron dependent, lipid peroxide accumulation‐induced nonapoptotic cell death.^27^ Previous studies indicated that ferroptosis was involved in the pathogenesis of many diseases, including acute lung injury, liver diseases, and kidney diseases.^8^ In recent years, the relationship between ferroptosis and endometritis has attracted extensive attention.^28^ A previous study has shown that nuciferine has a protective effect on endometritis by inhibiting ferroptosis.^29^ Also, citral has been known to inhibit endometritis through attenuating inflammation and ferroptosis.^10^ Our experiments demonstrated that CA treatment significantly attenuated ferroptosis induced by *S. aureus*. Currently, it is believed that multiple signalling pathways can regulate the occurrence of ferroptosis, among which the AMPK signalling pathway is one of its regulatory pathways.^30^ Activating this pathway can inhibit the occurrence and development of ferroptosis. Lee et al. found that ferroptosis is regulated by the AMPK signalling pathway and confirmed that activation of AMPK can inhibit the occurrence of ferroptosis.^31^ AMPK is a heterotrimeric protein kinase. The AMPK signalling pathway plays a crucial role in the human body, and various substances affect the body through this signalling pathway. Research has confirmed that the AMPK signalling pathway also plays an important role in LPS induced myocardial injury, and inhibiting AMPK can enhance cellular oxidative stress and inflammatory response.^32^ Activation of AMPK could regulate mTOR/HIF‐1α signalling pathway.^33^ In this study, we found CA significantly increased the expression of AMPK and inhibited HIF‐1α and phosphorylated mTOR expression. These data suggested that CA could regulate AMPKα/mTOR/HIF‐1α signalling pathway.

In summary, our experiments first showed CA inhibited endometritis through attenuating uterine pathological damage, inflammation, and ferroptosis. The mechanism was through regulating AMPKα/mTOR/HIF‐1α signalling pathway, which led to the inhibition of inflammation and ferroptosis.

## AUTHOR CONTRIBUTIONS

**Lu Cao:** Investigation (equal); methodology (equal); resources (equal); software (equal); writing – original draft (equal). **Junbao Liu:** Formal analysis (equal); investigation (equal); methodology (equal); supervision (equal); validation (equal); visualization (equal). **Cong Ye:** Conceptualization (equal); formal analysis (equal); funding acquisition (equal); investigation (equal); methodology (equal); software (equal). **Rui Qin:** Conceptualization (equal); investigation (equal); methodology (equal); software (equal); supervision (equal); validation (equal); writing – review and editing (equal). **Yubo Hu:** Writing – review and editing (equal).

## CONFLICT OF INTEREST STATEMENT

The authors have no relevant financial or non‐financial interests to disclose.

## CONSENT FOR PUBLICATION

All authors agree to publish in Journal of Cellular and Molecular Medicine.

## Data Availability

The data that support the findings of this study are available from the corresponding author upon reasonable request.
